# Microplastics increase susceptibility of amphibian larvae to the chytrid fungus *Batrachochytrium dendrobatidis*

**DOI:** 10.1038/s41598-021-01973-1

**Published:** 2021-11-17

**Authors:** Jaime Bosch, Barbora Thumsová, Naiara López-Rojo, Javier Pérez, Alberto Alonso, Matthew C. Fisher, Luz Boyero

**Affiliations:** 1 Biodiversity Research Institute, University of Oviedo-Principality of Asturias-CSIC, Mieres, Spain; 2 Centro de Investigación, Seguimiento y Evaluación, Parque Nacional Sierra de Guadarrama, Rascafría, Spain; 3 Museo Nacional de Ciencias Naturales-CSIC, Madrid, Spain; 4 Asociación Herpetológica Española, Madrid, Spain; 5 Department of Plant Biology and Ecology, University of the Basque Country (UPV/EHU), Leioa, Spain; 6 Laboratoire d’Ecologie Alpine (LECA), Université Grenoble Alpes, UMR CNRS-UGA-USMB, Grenoble, France; 7 MRC Centre for Global Infectious Disease Analysis, Department of Infectious Disease Epidemiology, Imperial School of Public Health, London, UK; 8 IKERBASQUE, Bilbao, Spain

**Keywords:** Invasive species, Environmental sciences

## Abstract

Microplastics (MPs), a new class of pollutants that pose a threat to aquatic biodiversity, are of increasing global concern. In tandem, the amphibian chytrid fungus *Batrachochytrium dendrobatidis* (*Bd*) causing the disease chytridiomycosis is emerging worldwide as a major stressor to amphibians. We here assess whether synergies exist between this infectious disease and MP pollution by mimicking natural contact of a highly susceptible species (midwife toads, *Alytes obstetricans*) with a *Bd-*infected reservoir species (fire salamanders, *Salamandra salamandra*) in the presence and absence of MPs. We found that MP ingestion increases the burden of infection by *Bd* in a dose-dependent manner. However, MPs accumulated to a greater extent in amphibians that were not exposed to *Bd*, likely due to *Bd*-damaged tadpole mouthparts interfering with MP ingestion. Our experimental approach showed compelling interactions between two emergent processes, chytridiomycosis and MP pollution, necessitating further research into potential synergies between these biotic and abiotic threats to amphibians.

## Introduction

Microplastics (MPs) are a widely emerging class of pollutants of global concern. They are plastic particles < 5 mm in size that originate either from primary (i.e., manufactured products) or secondary sources (i.e., fibres and fragments resulting from the breakdown of larger plastic items)^1,2^. Once in the environment, MPs accumulate due to their small size and resistance to biodegradation^3^. This class of pollutants are of growing concern due to their near ubiquity in marine^4^, freshwater^5^ and terrestrial ecosystems^6^ where they are increasingly being associated with negative health outcomes^7^. Despite mitigation strategies aimed at reducing plastic use and improving waste management, plastic pollution is becoming a major threat to the sustainability of our planet^8^.

The biological impacts of MPs are still poorly known, but evidence is accumulating that they interfere with core physiological processes including photosynthesis, food ingestion, metabolism, growth, and reproduction^9–12^. When external to the individual, MPs are known to interact with chemical pollutants, which could potentially increase the bioavailability of MPs or the organisms’ vulnerability to them^10,13,14^, as well as perhaps serving as physical vectors for the dispersal of pathogens^15,16^. However, despite their known impacts on biota, there is virtually no information about whether MPs influence the dynamics of infectious diseases in freshwater ecosystems^17^. This gap in our knowledge is especially relevant in the case of emerging infections of wildlife, where naïve hosts may have high susceptibility to new pathogens and for this reason require fully functioning anti-pathogen defences in order to survive.

A relevant system within which to explore potential interactions between infectious disease and MPs are amphibians and the aquatic chytrid fungus *Batrachochytrium dendrobatidis* (*Bd*). This fungus causes chytridiomycosis, a disease which is responsible for mass mortality and population declines of amphibians worldwide^18,19^. *Bd* likely originated in Asia in the early twentieth century, and the recent spread of its global pandemic lineage has been facilitated by humans^20^. As an ecological generalist pathogen, able to infect almost every amphibian species and other freshwater invertebrates, *Bd* has now invaded most areas of the world that contain amphibians^21^. However, the occurrence and severity of chytridiomycosis is context-dependent and determined by a complex interplay between biotic and abiotic factors that modulate the prevalence and intensity of infection caused by *Bd*^22,23^. Environmental stressors can trigger outbreaks of chytridiomycosis; these include climate warming^24^ and the presence of elevated concentrations of several contaminants such as metals^25^, tropospheric ozone^26^, antibiotics^27^ and pesticides^28,29^. Due to the growing burden of MPs in the water column, there is increasing potential for these pollutants to interact with *Bd* and its amphibian hosts in their aquatic environments to a greater or lesser extent. If interactions between the two do occur, then these synergies may alter the epidemiology of chytridiomycosis.

We examined the potential interaction between MP pollution and infection by *Bd* in the common midwife toad *Alytes obstetricans*. This species is broadly distributed across Europe and its tadpoles have long developmental periods that can exist for up to several years at high altitudes. Therefore, the exposure of midwife toad tadpoles to freshwater contaminants such as MPs and waterborne pathogens as *Bd* can be significant. Relevantly, this species has suffered severe declines caused by chytridiomycosis in certain regions of Europe^30,31^, and a previous study showed that increasing MP concentration progressively impaired their larval growth and body condition^32^. Here, we use an experimental approach that mimics natural interspecific spillover of *Bd* to investigate whether a rapidly emerging class of aquatic pollutants, MPs, have the potential to influence the epidemiology of chytridiomycosis. We hypothesized that common midwife toad larvae would be more susceptible to *Bd* in the presence of MPs, with infection prevalence and load being higher at higher MP concentrations. This hypothesis was tested in a laboratory experiment whereby larvae were exposed to different MP concentrations and *Bd* using a live co-infection model mimicking natural exposure to the pathogen.

## Results

**Figure 1:**
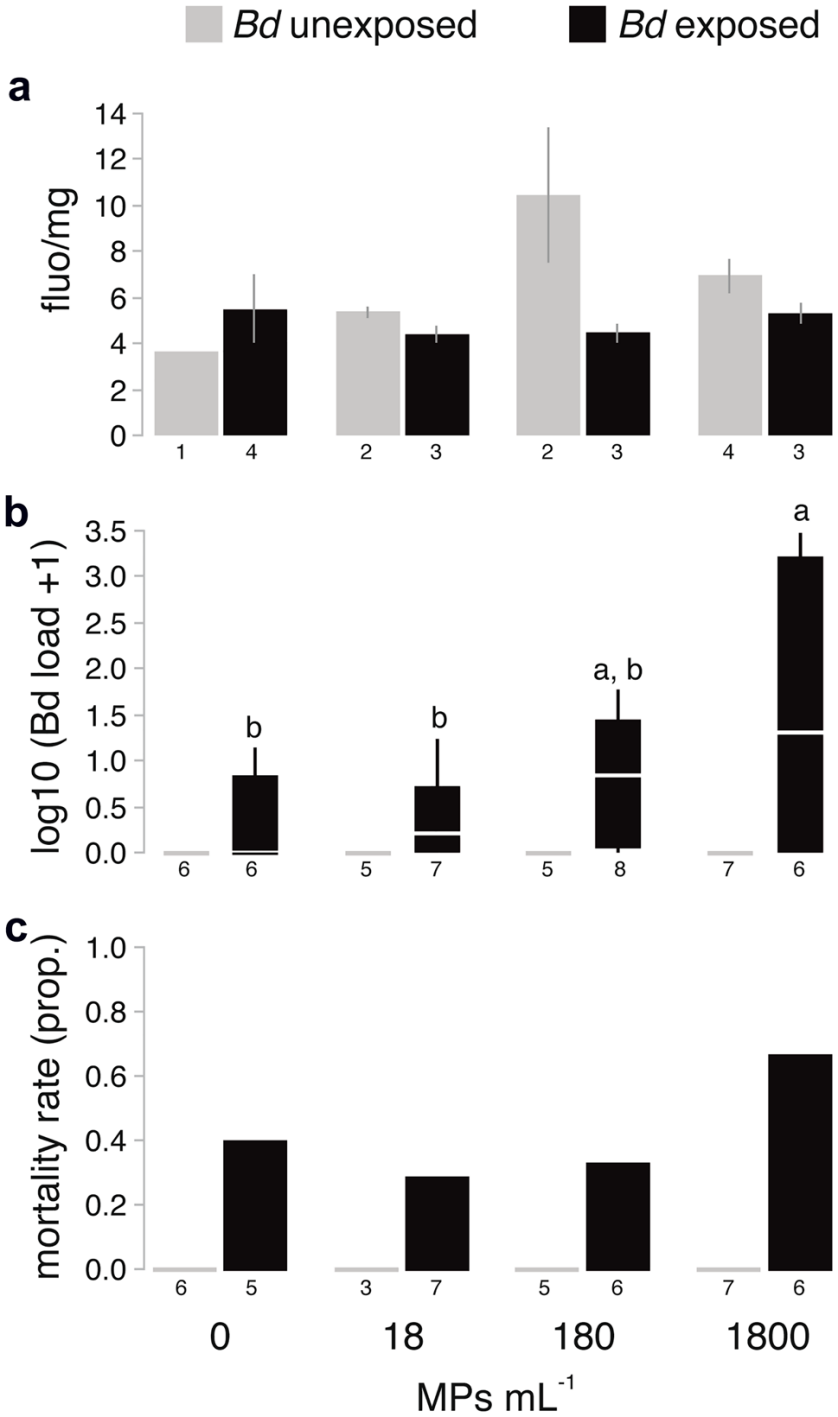
(**a**) Mean ± SE of fluorescence levels per tadpole mg, (**b**) log-transformed *Bd* loads (horizontal lines depict medians, boxes represent interquartile ranges and whiskers extend to minima-maxima), and (**c**) proportional tadpole mortality for each MP concentration (0, 18, 180 and 1800 MP mL^−1^) and *Bd* exposition treatment (*Bd* unexposed and *Bd* exposed). Numbers below bars indicate the number of replicates for each group, and different letters indicate statistically significant differences after post hoc Tukey tests (p < 0.05).

Fourteen out of 64 individuals remained as tadpoles at the end of the experiment at day 229 and were excluded for further analyses because *Bd* and MP treatments did not influence whether an individual underwent metamorphosis (χ^2^ = 4.79, df = 3, *p* > 0.1876). Levels of fluorescence due to MP presence, *Bd* loads, and rates of mortality amongst treatments are shown in Fig. 1 (raw data in Supplementary Table S1). Time to Gosner stage 42–43 ranged from 22 to 146 days and to reach Gosner stage 45 ranged from 34 to 158, and did not differ among treatments in any case (*p* > 0.4785), neither did body condition at metamorphosis (*p* > 0.2878). Mass at metamorphosis was dictated by tadpole mass when exposed to *Bd* (F_1,30_ = 42.16, *p* < 0.0001), but did not differ among treatments in any case (*p* > 0.2572). Levels of fluorescence did not vary among MP treatments (F_3,15_ = 0.79, *p* = 0.5190), and did not relate with *Bd* load (F_1,15_ = 1.01, *p* = 0.3324), but were significantly higher for tadpoles not exposed to *Bd* than for those exposed (F_1,15_ = 5.89, *p* = 0.0294).

All negative-control animals (no *Bd* exposure) were uninfected throughout the experiment. The proportion of infected animals at Gosner 42–43 did not differ across MP concentrations (0 mL^−1^: 33.3, 18: 57.1, 180: 75.0, 1800: 66.7%; χ^2^ = 2.78, df = 3, *p* = 0.4271) and was not influenced by tadpole body condition (χ^2^ = 0.48, df = 1, *p* = 0.4868). *Bd* load was not either influenced by tadpole body condition (F_1,41_ = 13.01, *p* = 0.0904). *Bd* load greatly differed between *Bd* treatments (F_1,41_ = 17.61, *p* = 0.0001) and also differed among MP treatments in a dose-dependent manner (F_3,41_ = 3.25, *p* = 0.0314), with a significant interaction term observed between *Bd* exposure and MP concentration (F_3,41_ = 3.17, *p* = 0.0341).

No mortalities were observed in any *Bd*-negative groups, but they were observed for the group of tadpoles exposed to *Bd* at the time of metamorphosis, and were greatest for the highest MP concentration (40.0, 28.6, 33.3 and 66.7% for 0, 18, 180, 1800 mL^−1^, respectively). However, the statistical model indicated that mortality was associated only with *Bd* load (χ^2^ = 4.19, df = 1, *p* = 0.0408), whereas the fixed factors and their interaction were not good predictors of mortality (*p* > 0.1416).

## Discussion

The emergence of *Bd* and chytridiomycosis worldwide is emblematic of the potential for endemic hotspots of infection to become globalised through anthropogenic processes^33^. Following invasion of the pathogen, a complex interplay among ecological factors determining disease outcome occurs including climate and altitude^34,35^, seasonality^35–38^, ultraviolet exposure^23,39^ and agrochemicals^40^ alongside numerous biotic modifiers^33^. These factors also include ecotoxicological influences, which include the emerging problem of microplastic (MP) pollution.

We took an in vivo experimental approach to measuring the accumulation of MPs through fluorescence. This showed that MPs accumulated to a greater extent in tadpoles that were not exposed to *Bd*. This finding could be explained by lower ingestion rates by tadpoles that were infected with *Bd*, given that tadpoles mainly ingest MPs when scraping on periphyton, where these particles are retained^32^. In tadpoles that were not exposed to *Bd*, MP ingestion increased up to concentrations of 180 part. mL^−1^ then exhibited a decrease, which is perhaps explained by high concentrations of MPs inhibiting feeding. This apparently non-linear effect was also seen when both *Bd* infection and MPs were combined, as ingestion appeared to also reduce feeding due to highly infected animals showing less MP accumulation that was accompanied by a slight loss of body condition. Here, the interaction may also have a component that is due to *Bd*-related damage to the tadpole mouthparts which may interfere with feeding^41^. Finally, it is also possible that MPs cause physical blockage or injury as has been shown for other organisms, leading to physiological changes in affected individuals^42^. These non-linear responses are likely ecologically relevant and further studies should focus on the physiological and mechanical consequences of MP accumulation and *Bd* infection on the feeding behaviour of *A. obstetricans* tadpoles.

For *Bd* exposed tadpoles, *Bd* load increased in line with MP concentration with only two values above 100 GE. Despite small sample sizes, we found that pathogen burden was highest at the highest concentration of MPs (1800 part. mL^−1^), lowest when MPs were most diluted (18 part. mL^−1^), and intermediate at the intermediate concentration (180 part. mL^−1^). This observation suggests that MP ingestion enhanced infection by *Bd* in a dose-dependent manner for a given level of exposure to the pathogen. Trends in mortality mirrored that of *Bd* load however were not significant and may be due again to the relatively small sample sizes used. At the time of writing, we do not know the causal relationship by which MPs lead to more intense *Bd* infections and attendant mortality. Future work needs to focus on the physiological changes that are experienced when tadpoles are exposed to MPs that may synergise with the immunological and physiological consequences of *Bd* exposure. For instance, infection by *Bd* in midwife toads is linked to changes in the glucocorticoid stress hormone, corticosterol^43^. If MPs cause generalised stress that increases levels of corticosterol, then this presents a pathway by which *Bd* infection could be linked to pollutant-exposure and can be explored.

We observed no mortality in animals in the *Bd* unexposed group indicating that MP exposure itself in this context was not lethal and no obvious losses in body condition were observed. This finding contrasts with a previous study showing high mortality in *A. obstetricans* tadpoles exposed to 1800 part. mL^−1^^32^. These differences could be due to the different larval stage used in both experiments: Boyero et al.^32^ used recently-hatched animals in comparison to the much more mature Gosner stages 26–36 that we used in this study. Smaller tadpoles are known to be more sensitive to pollutants^44^ and their metabolism is faster so MP ingestion was most likely higher in relation to body size. Experimental conditions also differed, with the microcosms used being smaller in Boyero et al.^32^ compared to those in our study, and MPs were from a different commercial source. For this reason, our experimental findings are likely conservative, and longer-term exposure of tadpoles to MPs in synergy with *Bd* exposure may well result in more aggressive patterns of mortality as health-costs accrue through the animals’ development.

More widely, studies have shown that the joint effects of different stressors on biota in freshwater ecosystems are antagonistic^45^. Here, we have shown an interaction between two anthropogenically associated stressors, a pathogen and a pollutant, and argue that future work needs to explore more complex interactions between amphibian health and their changing environments. Importantly, amphibian larvae coexist with naturally occurring micropredators, such as protists, rotifers and crustaceans. These plankton are not only capable of using *Bd* zoospores as a food source^46^ but may also sequester MPs from the water column. The extent to which this occurs, and whether it exacerbates or mitigates the burden of infection, is uncertain but deserves to be explored. Certainly, the influx of MPs into aquatic environments will perturb the structure of foodwebs, with expected, but unknown and currently unquantified knock-on effects on host–pathogen dynamics. Here, we have shown that an interaction between *Bd* and its amphibian host within the context of emerging MP pollution is an alarming aspect of these animals’ rapidly changing world.

## Methods

### Experimental procedure

The experiment was conducted under biosecure conditions at the laboratories of the Research and Management Center ‘Puente del Perdón’. This facility, located at the Sierra de Guadarrama National Park (Spain), maintains a colony of *A. obstetricans* for reintroduction purposes. Sixty-four 42 × 30 × 10 cm tanks were filled with 2 L of well water and MPs at different concentrations. MPs were 10 μm green fluorescent (508/542 nm) polystyrene microspheres (Thermo Scientific™ Fluoro-Max™ Fluorescent beads) internally dyed to emit bright and intense colour when illuminated by fluorescent light (excitation/emission peaks of 468/508 nm), suspended in aqueous solution [1.8 × 10^7^ particles mL^−1^, 1% solids, density = 1.05 g/cm^3^, refraction index = 1.59 @ 589 nm (25 °C), trace amount (< 0.05%) of the surfactant sodium azide]. Following Boyero et al.^32^, we used four dilutions of MP with final concentrations of 0, 18, 180 and 1800 part. mL^−1^. These concentrations are known to cover exposures that have effects on amphibian tadpoles that range from neutral through to highly deleterious^32^. Each solution was sonicated at 50 kHz for 5 min to suspend particles before being introduced to each tank.

We used 64 *Bd*-free tadpoles of *A. obstetricans* born in captivity at the above-mentioned facilities. Most tadpoles (89%) were in development Gosner stages 26, whereas just seven animals differed in their Gosner stages (ranging from 32 to 36). Tadpoles were individually added to each tank containing final MP concentrations on the same day, where they were fed ad libitum with commercial spirulina tabs (SERA GmbH, Heinsberg, Germany) during the experiment. In order to obtain the two *Bd* treatments (*Bd*-positive and *Bd*-negative), we collected 30 overwintered *Salamandra salamandra* larvae from a permanent pond and 30 non-overwintered *S. salamandra* larvae from a temporary pond at the Sierra de Guadarrama National Park. Prevalence of *Bd* infection of overwintered salamander larvae from the permanent pond is known to approach 100% in early spring, whereas it is 0% at the temporary pond^47^. Salamander larvae from both locations were separately acclimated for 48 h in two 20 L tanks at 16 °C. The bodies of 20 specimens of each group were swabbed following a standardized protocol^48^ to confirm their infection status by quantitative polymerase chain reaction (qPCR, see below). Eight *A. obstetricans* tadpoles of each experimental MP group were randomly assigned to the *Bd*-positive treatment group, whereas the remaining eight tadpoles were assigned to the *Bd*-negative treatment group. Starting at day 78 of MP exposure, and for six consecutive days, tadpoles at the *Bd*-positive group received daily 500 μL of water from the *Bd*-positive salamander tank, whereas the other tadpoles received 500 μL of water from the *Bd*-negative salamander tank. Water from salamander tanks was pipetted directly onto the dorsum of each tadpole. Prior to the *Bd* exposure, all *A. obstetricans* tadpoles were individually measured (total length, precision of 0.01 mm) and weighed (precision of 0.01 g).

The experiment ran for 229 days, with medium replacement (i.e., water or MP solutions) every 14 days, and inspection for metamorphosis or potential mortality every second day. Average water temperature during the course of the experiment was 13.5 °C (± 2.7 SD), and animals were reared on a 12:12 h natural light cycle and fed ad libitum with spirulina (tadpoles) or with baby crickets (after metamorphosis). A toe-clip of each individual was taken and preserved in 70% ethanol when they reached Gosner 42–43 (i.e., forelimbs had emerged and mouthparts had been restructured for terrestrial foraging). At Gosner 45 (i.e., the tail had receded to a stub), the water in the tank was reduced by 90% and individuals were measured (snout-to-vent length, SVL), weighed and observed for lethal chytridiomycosis during the next 15 days. Dead individuals were collected, digested for a minimum of seven days in H_2_O_2_ (30% v/v) and processed for MP examination with fluorescence following Boyero et al.^32^, adapted from Hu et al.^49^. When there was no mortality within an experimental group, two randomly selected individuals were euthanized (MS-222 100 mg L^−1^, 7.5 pH buffered with NaHCO_3_) for MP examination.

The animal experiment complied with the ARRIVE guidelines, was carried out in accordance with the EU Directive 2010/63/EU for animal experiments and was approved by ‘Comité de Ética del MNCN-CSIC’.

### qPCR analyses

DNA was extracted using PrepMan Ultra reagent, and extractions were diluted 1:10 before qPCR amplification per duplicate following Boyle et al.^50^ by using a myGo Pro qPCR machine. Negative controls and standards with known concentrations of *Bd* (0.1, 1, 10 and 100 genomic equivalents of zoospores, here referred to as GE) were used in each plate. Infection load of each sample was assessed directly by the machine software according to the reference function obtained with the standards of known concentration of zoospore. A sample was considered positive when its infection load was equal to or higher than 0.1 GE, and the amplification curve presented a robust sigmoidal shape.

### Microplastic examination by fluorescence

MP examination was performed by quantifying fluorescence of digested samples using a fluorimeter (GENios™ plate reader, TECAN; Durham, NC, USA) at an excitation wavelength of 485 nm and emission wavelength of 535 nm. Digested samples were all filled to 12 mL and sonicated (35 Hz, 3 min) before being transferred into a 96-well plate, which included all the samples (3 replicates each) and distilled water solutions with known MP concentrations (0, 180, 900, 1800, 9000 and 18,000 part. mL^−1^; 4 replicates each). Fluorescence measures were corrected by tadpole weight at Gosner 45.

### Data analyses

ANOVA tests and post hoc Tukey tests were used to assess statistically significant differences among MP and *Bd* treatments for the following continuous independent variables after being log10(x + 1)-transformed to reach normality: ‘time to reach Gosner stage 42–43’ and ‘time to reach Gosner 45’, ‘mass’, ‘body condition’ and ‘fluorescence levels at Gosner 45’, and ‘*Bd* loads at Gosner 42–43’. In all cases, MP concentration and *Bd* exposure were used as fixed factors, with their interaction included. ‘Tadpole body condition’, calculated following Peig and Green^51^, was introduced as a covariate in the ANOVA tests for ‘*Bd* load’ and ‘time to reach Gosner stage 42–43 and Gosner 45’. ‘Tadpole mass when exposed to *Bd*’ was introduced as a covariate in the ANOVA test for ‘mass at Gosner 45’. ‘*Bd* load at Gosner 42–43’ was introduced as a covariate in the ANOVA test for ‘fluorescence levels at Gosner 45’.

Generalized linear models with binomial errors were used for the binomial independent variables ‘*Bd* status’, ‘mortality’ and ‘reached metamorphosis’ at the latest stages of the experiment to assess statistically significant differences among MP and *Bd* treatments. MP concentration and *Bd* exposure were used as fixed factors, with their interaction included, except for ‘*Bd* status at the end of the experiment’ because none of the non-*Bd* exposed individual tested positive for *Bd*. ‘Tadpole body condition’, and ‘*Bd* loads at Gosner 42–43’ were introduced as covariates to asses if MP and *Bd* treatments had any effect on whether an individual undergoes metamorphosis, or died at the end of the metamorphosis, respectively.

‘Development stage’ was not included as a covariate in the statistical models because preliminary analyses discarded any significant contribution of this variable in any case. All statistical analyses were performed with JMP Pro 12 (SAS Inc.).

## Supplementary Information

Supplementary Table S1.
