# Supplementary material for: Microplastics increase susceptibility of amphibian larvae to the chytrid fungus Batrachochytrium dendrobatidis
Source: Sci Rep. 2021 Nov 17;11:22438. doi: 10.1038/s41598-021-01973-1 (PMC8599647; doi:10.1038/s41598-021-01973-1)
Supplement: Supplementary file 1 — Supplementary Table S1. [file 41598_2021_1973_MOESM1_ESM.pdf]

Microplastics increase susceptibility of amphibian larvae to the chytrid fungus *Batrachochytrium dendrobatidis*

Jaime Bosch*[1,2,3], Barbora Thumsová[3,4], Naiara López-Rojo[5,6], Javier Pérez[5], Alberto Alonso[5], Matthew C. Fisher[7], Luz Boyero[5,8]

[1] Biodiversity Research Institute, University of Oviedo-Principality of Asturias-CSIC, Mieres, Spain.

[2] Centro de Investigación, Seguimiento y Evaluación, Parque Nacional Sierra de Guadarrama, Rascafría, Spain.

[3] Museo Nacional de Ciencias Naturales-CSIC, Madrid, Spain.

[4] Asociación Herpetológica Española, Madrid, Spain.

[5] Department of Plant Biology and Ecology, University of the Basque Country (UPV/EHU), Leioa, Spain.

[6] Laboratoire d'Ecologie Alpine (LECA), Université Grenoble Alpes, UMR CNRS-UGA-USMB, Grenoble, France.

[7] MRC Centre for Global Infectious Disease Analysis, Department of Infectious Disease Epidemiology, Imperial School of Public Health, London, UK.

[8] IKERBASQUE, Bilbao, Spain.

*Corresponding author: jaime.bosch@csic.es, +34677772402

Supplementary Table S1. Raw data on the experimental animals.
NA: data not available.

individual	Bd treatment	MP treatment (MPs mL-1)	tadpole Gosner stage	tadpole total length (mm)	tadpole weight (g)	tadpole body condition	time to reach Gosner stage 42–43 (days)	Bd load at Gosner stage 42–43 (GE)	remained as tadpole?	time to reach metamorphosis (days)	mortality at metamorphosis?	weight at metamorphosis (g)	SVL at metamorphosis (mm)	body condition at metamorphosis	fluorescence	MP/ml
A1	Bd unexposed	0	36	69.4	1.80	1.130	28	0	no	43	no	1.05	23	0.701	3808	67
A2	Bd unexposed	0	26	41.5	0.43	1.130	NA	NA	yes	NA	NA	NA	NA	NA	NA	NA
A3	Bd unexposed	0	26	60.6	1.00	0.910	63	0	no	70	no	0.80	20	0.789	NA	NA
A4	Bd unexposed	0	26	63.9	1.25	0.980	43	0	no	57	no	0.70	20	0.691	NA	NA
A5	Bd unexposed	0	26	59.9	1.25	1.180	63	0	no	70	no	0.75	19	0.854	NA	NA
A6	Bd unexposed	0	26	63.0	1.30	1.060	57	0	no	63	no	0.80	19	0.911	NA	NA
A7	Bd unexposed	0	26	41.9	0.35	0.900	NA	NA	yes	NA	NA	NA	NA	NA	NA	NA
A8	Bd unexposed	0	26	67.6	1.45	0.970	57	0	no	63	no	1.00	22	0.756	32168	12790
B1	Bd unexposed	18	26	68.2	1.45	0.950	43	0	no	57	no	0.85	21	0.732	NA	NA
B2	Bd unexposed	18	26	64.0	1.25	0.980	43	0	no	NA	NA	NA	NA	NA	NA	NA
B3	Bd unexposed	18	26	57.8	0.95	0.990	93	0	no	97	no	0.80	19	0.911	4124	209
B5	Bd unexposed	18	26	56.0	1.00	1.140	NA	NA	yes	NA	NA	NA	NA	NA	NA	NA
B6	Bd unexposed	18	26	65.3	1.20	0.890	22	0	no	43	no	0.85	20	0.839	4788	507
B7	Bd unexposed	18	26	53.1	0.70	0.920	NA	NA	yes	NA	NA	NA	NA	NA	NA	NA
B8	Bd unexposed	18	26	43.0	0.35	0.830	NA	NA	yes	NA	NA	NA	NA	NA	NA	NA
B9	Bd unexposed	18	32	68.2	1.60	1.050	43	0	no	NA	NA	NA	NA	NA	NA	NA
C1	Bd unexposed	180	33	69.4	1.65	1.030	28	0	no	43	no	0.50	22	0.378	3789	59
C2	Bd unexposed	180	26	59.3	1.10	1.070	57	0	no	63	no	0.70	19	0.797	NA	NA
C4	Bd unexposed	180	26	65.4	1.50	1.110	22	0	no	43	no	0.85	21	0.732	11519	3527
C5	Bd unexposed	180	26	64.4	1.35	1.040	22	0	no	43	no	0.70	21	0.603	NA	NA
C6	Bd unexposed	180	26	62.2	1.20	1.020	57	0	no	63	no	0.80	21	0.689	NA	NA
C7	Bd unexposed	180	26	52.2	0.75	1.040	NA	NA	yes	NA	NA	NA	NA	NA	NA	NA
C8	Bd unexposed	180	26	47.3	0.55	1.000	NA	NA	yes	NA	NA	NA	NA	NA	NA	NA
C9	Bd unexposed	180	26	50.1	0.70	1.090	NA	NA	yes	NA	NA	NA	NA	NA	NA	NA
D1	Bd unexposed	1800	26	51.5	0.65	0.930	NA	NA	yes	NA	NA	NA	NA	NA	NA	NA
D2	Bd unexposed	1800	26	51.7	0.70	1.000	93	0	no	93	no	0.50	18	0.662	4244	263
D3	Bd unexposed	1800	35	63.1	1.30	1.060	22	0	no	43	no	0.60	20	0.592	NA	NA
D4	Bd unexposed	1800	26	61.4	1.30	1.140	43	0	no	57	no	0.75	20	0.740	4324	299
D6	Bd unexposed	1800	26	55.2	0.90	1.070	93	0	no	97	no	0.50	18	0.662	NA	NA
D7	Bd unexposed	1800	26	69.0	1.40	0.890	43	0	no	57	no	0.85	20	0.839	4692	464
D8	Bd unexposed	1800	26	56.9	0.90	0.980	70	0	no	76	no	0.45	17	0.699	NA	NA
D9	Bd unexposed	1800	26	56.5	0.85	0.940	113	0	no	116	no	0.50	21	0.430	4087	193
E1	Bd exposed	0	26	44.5	0.50	1.080	146	0	no	NA	NA	NA	NA	NA	NA	NA
E2	Bd exposed	0	36	72.1	1.70	0.950	28	5	no	43	yes	1.00	19	1.139	3792	60
E3	Bd exposed	0	26	50.0	0.65	1.010	NA	NA	yes	NA	NA	NA	NA	NA	NA	NA
E4	Bd exposed	0	26	48.4	0.65	1.120	NA	NA	yes	NA	NA	NA	NA	NA	NA	NA
E5	Bd exposed	0	26	64.6	1.40	1.070	22	0	no	43	no	0.95	21	0.818	3624	-15
E6	Bd exposed	0	26	67.0	1.50	1.030	57	0	no	63	no	0.90	21	0.775	NA	NA
E7	Bd exposed	0	26	69.5	1.70	1.060	43	0	no	57	yes	0.85	22	0.643	3762	47
E8	Bd exposed	0	26	59.2	0.95	0.930	113	13	no	116	no	0.40	20	0.395	4043	173
F1	Bd exposed	18	26	65.4	1.25	0.920	28	16	no	NA	yes	NA	22	NA	3920	118
F2	Bd exposed	18	26	50.8	0.65	0.970	97	0	no	113	no	0.40	16	0.736	NA	NA
F4	Bd exposed	18	26	46.2	0.40	0.780	NA	NA	yes	NA	NA	NA	NA	NA	NA	NA
F5	Bd exposed	18	26	59.6	1.00	0.960	43	1	no	57	yes	0.90	22	0.680	3754	43
F6	Bd exposed	18	26	43.5	0.45	1.040	97	4	no	NA	no	NA	NA	0.661	NA	NA
F7	Bd exposed	18	26	69.2	1.70	1.070	22	0	no	43	no	1.00	22	0.756	NA	NA
F8	Bd exposed	18	35	70.3	1.45	0.870	22	0	no	43	no	0.95	21	0.818	3722	29
F9	Bd exposed	18	26	53.8	0.75	0.960	93	1	no	113	no	0.40	16	0.736	NA	NA
G1	Bd exposed	180	26	55.4	0.90	1.060	63	1	no	70	no	0.50	18	0.662	NA	NA
G2	Bd exposed	180	26	62.1	1.30	1.110	28	13	no	NA	yes	1.05	22	0.794	3892	105
G3	Bd exposed	180	26	59.3	1.10	1.070	57	35	no	63	no	0.80	20	0.789	4064	182
G4	Bd exposed	180	26	58.7	0.95	0.950	93	0	no	NA	NA	NA	NA	NA	NA	NA
G5	Bd exposed	180	36	67.7	1.65	1.110	28	5	no	43	yes	0.85	20	0.839	3949	130
G6	Bd exposed	180	26	59.5	1.00	0.960	57	58	no	63	no	0.70	19	0.797	NA	NA
G7	Bd exposed	180	26	54.3	0.75	0.930	93	7	no	NA	no	NA	NA	0.592	NA	NA
G8	Bd exposed	180	26	59.4	0.85	0.820	146	0	no	NA	NA	NA	NA	NA	NA	NA
H1	Bd exposed	1800	26	56.2	0.90	1.020	NA	NA	yes	NA	NA	NA	NA	NA	NA	NA
H2	Bd exposed	1800	26	51.1	0.85	1.250	70	0	no	76	no	0.35	17	0.544	NA	NA
H4	Bd exposed	1800	35	70.5	1.90	1.130	22	0	no	43	no	0.95	22	0.718	NA	NA
H5	Bd exposed	1800	26	61.7	1.25	1.080	43	58	no	NA	yes	NA	NA	NA	4261	271
H6	Bd exposed	1800	26	65.9	1.30	0.940	NA	2963	no	NA	yes	NA	NA	NA	4707	471
H7	Bd exposed	1800	26	50.2	0.75	1.160	NA	NA	yes	NA	NA	NA	NA	NA	NA	NA
H8	Bd exposed	1800	26	60.8	1.15	1.040	43	6	no	57	yes	0.85	20	0.839	3761	46
H9	Bd exposed	1800	26	70.8	1.80	1.060	43	1323	no	NA	yes	NA	NA	0.701	NA	NA
